# Spinal CSF flow in response to forced thoracic and abdominal respiration

**DOI:** 10.1186/s12987-019-0130-0

**Published:** 2019-04-04

**Authors:** Gökmen Aktas, Jost M. Kollmeier, Arun A. Joseph, Klaus-Dietmar Merboldt, Hans-Christoph Ludwig, Jutta Gärtner, Jens Frahm, Steffi Dreha-Kulaczewski

**Affiliations:** 1 School of Medicine, University Medical Center Göttingen, 37075 Göttingen, Germany; 2 Biomedizinische NMR, Max-Planck-Institut für biophysikalische Chemie, 37077 Göttingen, Germany; 3 DZHK (German Center for Cardiovascular Research), Partner Site Göttingen, Germany; 4 Division of Pediatric Neurosurgery, Department of Neurosurgery, University Medical Center Göttingen, 37075 Göttingen, Germany; 5 Division of Pediatric Neurology, Department of Pediatrics and Adolescent Medicine, University Medical Center Göttingen, 37075 Göttingen, Germany

**Keywords:** Flow-sensitive real-time MRI, CSF dynamics, Respiration, Hydrocephalus, Intrathoracic pressure, Intraabdominal pressure

## Abstract

**Background:**

Respiration-induced pressure changes represent a powerful driving force of CSF dynamics as previously demonstrated using flow-sensitive real-time magnetic resonance imaging (MRI). The purpose of the present study was to elucidate the sensitivity of CSF flow along the spinal canal to forced thoracic versus abdominal respiration.

**Methods:**

Eighteen subjects without known illness were studied using real-time phase-contrast flow MRI at 3 T in the aqueduct and along the spinal canal at levels C3, Th1, Th8 and L3. Subjects performed a protocol of forced breathing comprising four cycles of 2.5 s inspiration and 2.5 s expiration.

**Results:**

The quantitative results for spinal CSF flow rates and volumes confirm previous findings of an upward movement during forced inspiration and reversed downward flow during subsequent exhalation—for both breathing types. However, the effects were more pronounced for abdominal than for thoracic breathing, in particular at spinal levels Th8 and L3. In general, CSF net flow volumes were very similar for both breathing conditions pointing upwards in all locations.

**Conclusions:**

Spinal CSF dynamics are sensitive to varying respiratory performances. The different CSF flow volumes in response to deep thoracic versus abdominal breathing reflect instantaneous adjustments of intrathoracic and intraabdominal pressure, respectively. Real-time MRI access to CSF flow in response to defined respiration patterns will be of clinical importance for patients with disturbed CSF circulation like hydrocephalus, pseudotumor cerebri and others.

**Electronic supplementary material:**

The online version of this article (10.1186/s12987-019-0130-0) contains supplementary material, which is available to authorized users.

## Background

The human CSF system consists of the brain ventricular system and outer subarachnoid spaces which expand between the outer brain surface and skull. Free communication with spinal subarachnoid spaces takes place at the cranio-cervical junction, the large aperture (foramen magnum) between skull and spinal canal. Because the aqueduct interconnects the 4th and 3rd ventricle within the brain, CSF exchange between supratentorial ventricles and outer subarachnoid spaces exclusively passes through the aqueduct.

Cerebrospinal fluid flow has been thought to mainly follow cardiac-related oscillations as suggested by electrocardiogram (ECG)-synchronized cine flow magnetic resonance imaging (MRI) [1, 2]. In contrast, flow MRI techniques without experimental prejudice due to cardiac gating revealed the significant influence of respiration [3–7]. In particular, forced inspiration has been identified as the dominant regulator of CSF dynamics in all its compartments using flow-sensitive real-time MRI, while flow adjustments in relation to the heart beat represent a continuous albeit minor component [8, 9]. Forced inhalation prompted an upward surge of CSF from the thecal sac in the lumbar region along the entire spinal canal, into the cranial vault and passing through the aqueduct further upwards [10].

The upward motion of CSF into the head and brain is explained by the necessity to counterbalance inspiratory-regulated venous outflow out of the head/neck region [9]. The interplay between the CSF and venous blood system is part of a tightly adjusted fluid equilibrium essential to ascertain a constant intracranial volume in accordance with the Monro-Kellie doctrine [11]. In the spinal canal concomitant forced expiration revealed downward CSF flow which resulted in a watershed pattern with the dividing point at about the level of the heart. Upward direction prevailed cranial to thoracic level Th1, while CSF flow pointed downwards at level Th6 and below.

Human physiology discriminates two types of respiration, i.e. abdominal and thoracic breathing. Thoracic breathing, usually considered more shallow, mainly involves the muscles of the bony thoracic cage. Abdominal breathing is primarily defined by movements of the diaphragm [12]. Both breathing types provoke pressure changes in the abdominal and thoracic cavity, respectively, albeit to a different extent [13]. For example, Kaneko et al. [14] found that the abdomen in general yields greater breathing movements than the thorax.

Cerebrospinal fluid properties like pressure and volume have been reported to adapt to abrupt changes of abdominal and thoracic pressures like coughing as well as to long-lasting alterations in obesity either within the whole fluid system or locally in the lumbosacral region [15, 16].

The current real-time flow MRI study focused on the responsiveness of CSF dynamics to differences between abdominal versus thoracic breathing and their concomitant pressure changes. To account for possible regional differences the entire CSF system from the lumbar region to the brain aqueduct was covered.

## Methods

### Subjects

Eighteen subjects (9 females, age range 18–31 years, 23.9 ± 3.2; mean ± SD; height 176.8 ± 8.7 cm, weight 76.6 ± 14.8 kg, body mass index 24.3 ± 18.5 kg m^−2^; mean ± SD) with no known illness, in particular without pulmonary ailment or contraindication for MRI, were recruited. The study was approved by the institutional review board and written informed consent was obtained from each subject prior to MRI. The study was in compliance with the Declaration of Helsinki.

### Study design

**Fig. 1:**
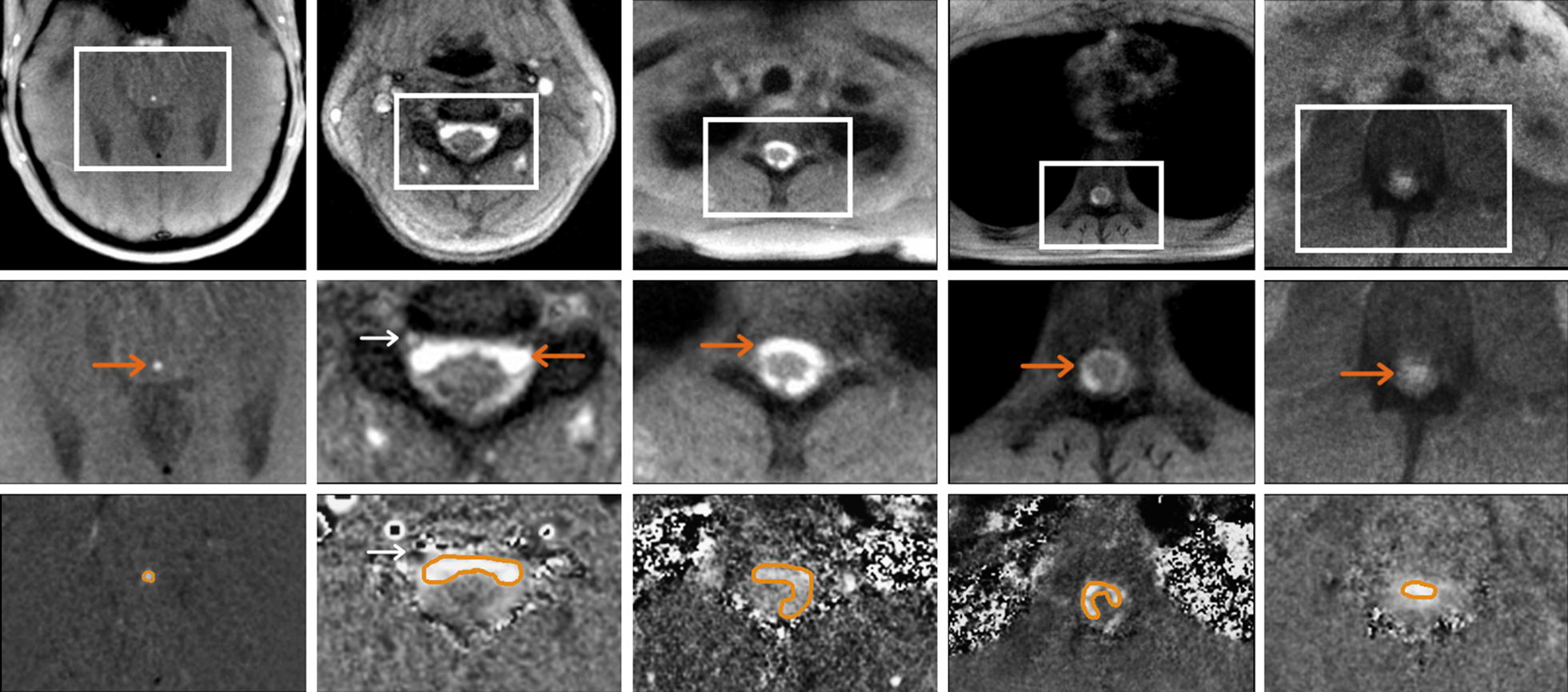
Regions of interest for CSF flow analyses. Top: magnitude images and Middle: magnified sections reveal CSF flow (bright signals, orange arrows ) during forced inspiration in (left to right) the aqueduct as well as at spinal level C3, Th1, Th8 and L3 of one representative subject (#7). Bottom: corresponding magnified velocity maps indicate upward flow during forced inspiration (bright signals, orange ROI), whereas epidural veins at spinal level C3 (white arrows) show concomitant downward flow of venous blood out of the head

Five locations were selected for the analysis of flow MRI data (see Fig. 1 and Additional file 1: Figure S1). The subarachnoid spinal CSF spaces were covered along the spinal canal at lumbar level L3, thoracic levels Th8 and Th1, and cervical level C3. A further region-of-interest (ROI) was placed in the aqueduct to link to the CSF dynamics in the brain ventricles.

Prior to MRI, subjects were instructed and trained in thoracic and abdominal breathing as well as in the timing of the predefined breathing protocol. Once placed in the scanner in supine position, they were required to follow visually presented commands during data acquisition: a starting phase with 10 s of normal breathing followed by four cycles of 2.5 s forced inspiration and 2.5 s forced expiration, and again 10 s of normal breathing (see Additional file 2: Figure S2). Each location was studied twice, while subjects performed first thoracic breathing followed by abdominal breathing. Adherence to the protocol was verified by comparing the movements of the thoracic and abdominal walls with the timing of the visual guiding protocol (see Additional file 2: Figure S2). All subjects were monitored via ECG and a respiration belt at about the level of the diaphragm. The breathing performances of the subjects in the scanner were evaluated by observation and measurements were repeated if necessary.

### Real-time MRI

Real-time phase-contrast flow MRI was performed at 3 T (Magnetom Prisma Fit; Siemens Healthcare). The technique is based on flow-encoded radial FLASH acquisitions with pronounced data undersampling and image reconstruction by nonlinear inversion and offers access to high spatial and temporal resolution [17–20]. For this study, the sequence exploited ideas by Bernstein et al. [21] for minimizing the gradient-echo time (TE) of flow-encoded acquisitions. The scan parameters were as follows: repetition time (TR) 5.68 ms, TE 4.61 ms, slice thickness 5 mm, flip angle 10°. The field of view was 192 mm or 256 mm depending on the position along the spine, while the in-plane resolution was fixed to 0.75 × 0.75 mm^2^. Two flow-encoded acquisitions were each acquired with 11 radial spokes yielding a temporal resolution of 125 ms per phase-contrast velocity map. The velocity sensitivity varied between 10 and 40 cm s^−1^ depending on the expected flow velocities and the breathing performance of the subjects. Lumbar (L3) and thoracic regions (Th8 and Th1) of the spinal canal were measured using suitable elements of the 18-channel thorax coil and 32-channel spine coil, while acquisitions at the cervical spinal canal (C3) and aqueduct were conducted with use of the 64-channel head coil.

**Fig. 2:**
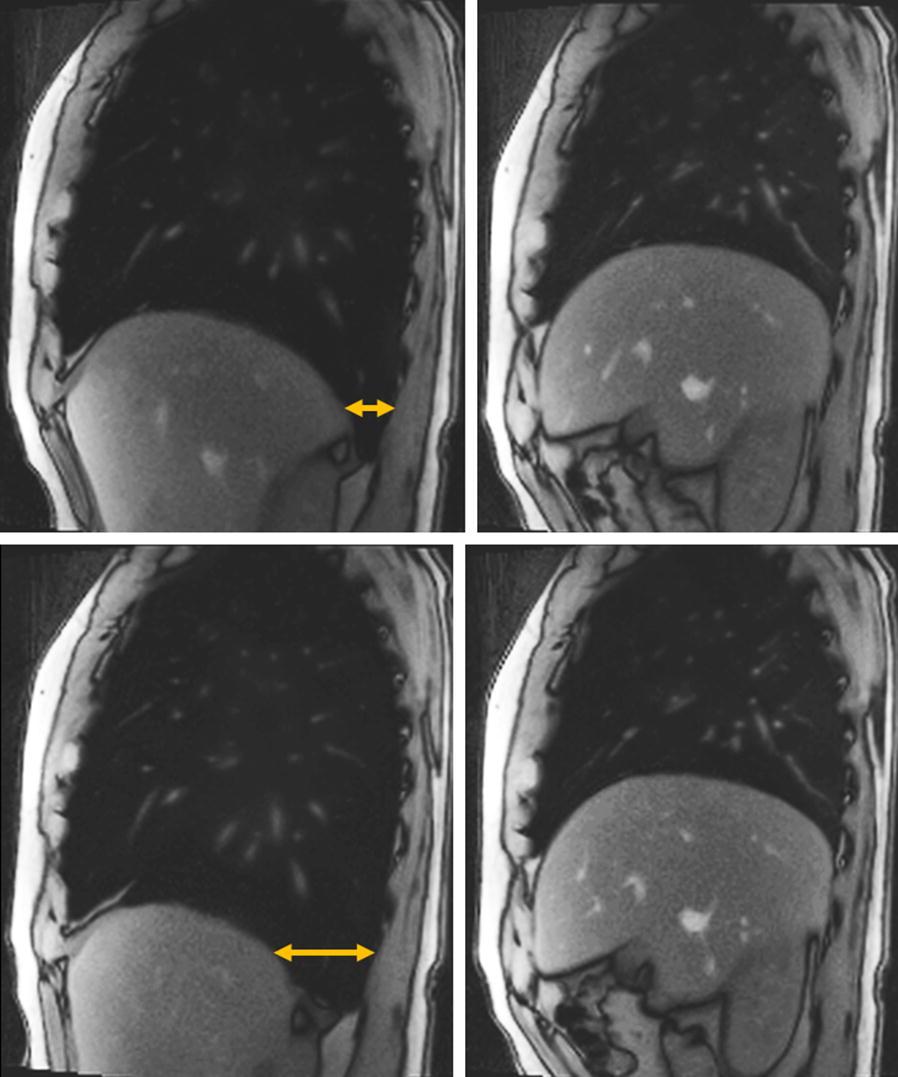
Real-time MRI of thoracic and abdominal breathing. Selected sagittal real-time images through the right dome of the diaphragm during thoracic and abdominal respiration. Upper left: thoracic breathing at deep inspiration and upper right: deep expiration. Lower left: abdominal breathing at deep inspiration and lower right: deep expiration. Inspiration causes elevation of ribcage, widening of anteroposterior thoracic diameters, contraction of the diaphragm with downward movement of its dome and enlargement of the intrathoracic volume. The corresponding widening of the costodiaphragmatic recess (arrows) is more pronounced during abdominal inspiration

Real-time phase-contrast MRI data, i.e. magnitude images and corresponding velocity maps, were reconstructed online at a rate of about 3.5 frames per second (fps). This performance required a highly parallelized version of the reconstruction algorithm [22] and its implementation on a bypass computer (Sysgen, Bremen, Germany) to the host computer of the MRI system consisting of two processors (SandyBridge E5-2650, Intel) and eight graphical processing units (GeForce GTX TITAN, Nvidia). Anatomical real-time images of thoracic and abdominal breathing movements (see Fig. 2) were obtained at a rate of 30 fps using the following parameters: TR 1.96 ms, TE 1.22 ms, slice thickness 6 mm, flip angle 8°, field of view 320 mm, 1.6 × 1.6 mm^2^ resolution, 17 radial spokes, and 33.3 ms temporal resolution.

### Data analysis

Qualitative and quantitative analyses of real-time flow MRI measurements were performed using CaFuR prototype software (Fraunhofer Mevis, Bremen, Germany) especially designed to accomplish automatic segmentation of flow signals in real-time image series [23]. Manual definition of an initial ROI for the determination of through-plane CSF flow was based on both signal intensities in magnitude images and corresponding phase values (i.e., velocities) in phase-contrast maps (see Fig. 1 lower row for representative examples). Further processing of data was performed using Matlab (Mathworks, USA).

Deviations of the subjects’ breathing performance from the visually instructed protocol were corrected for by shifting the acquired flow rates (ml s^−1^) in time. This is because the calculation of flow volumes per respiratory phase from the measured flow profile might lead to false results when using a breathing protocol with fixed timing which does not correspond to the actual performance. The time shift for the entire curve was obtained using a simple cross-correlation analysis which leads to a “best match” of the actual breathing performance to the requested protocol (see Additional file 2: Figure S2, subject #18 at L3). The correction was only applied to flow profiles with normalized correlation coefficients larger than 1/3: other cases were not considered reliable enough. The efficiency of the strategy was further controlled by a direct comparison to the respiration-induced movements of the abdominal or chest wall which are detectable in the serial magnitude images (see Additional file 2: Figure S2).

## Results

Figure 1 shows original (top) and zoomed magnitude images (middle) as well as corresponding zoomed velocity maps (bottom) during forced inspiration (selected from real-time flow MRI acquisitions) at all five locations of one representative subject (#7). Magnitude images exhibit a high sensitivity to through-plane flow because the inflow of unsaturated spins increases respective signal intensities. On the other hand, dark and bright signals in velocity maps correspond to flow directionality with grey values representing zero movement. During inspiration the occurrence of bright signals refers to upward flow, while simultaneous dark signals represent downward flow in epidural veins where fluid movement is opposite to that of CSF.

### CSF flow and breathing type

The marked difference between thoracic and abdominal breathing is illustrated in sagittal views of the thorax in Fig. 2 as well as Additional file 3: Video S1 and Additional file 4: Video S2 using anatomical real-time MRI at 30 fps. The movement of the diaphragm is more pronounced during forced abdominal than thoracic inspiration as demonstrated by the enlarged opening of the costodiaphragmatic recess, i.e. the space between diaphragm and rear wall of the rib cage.

**Fig. 3:**
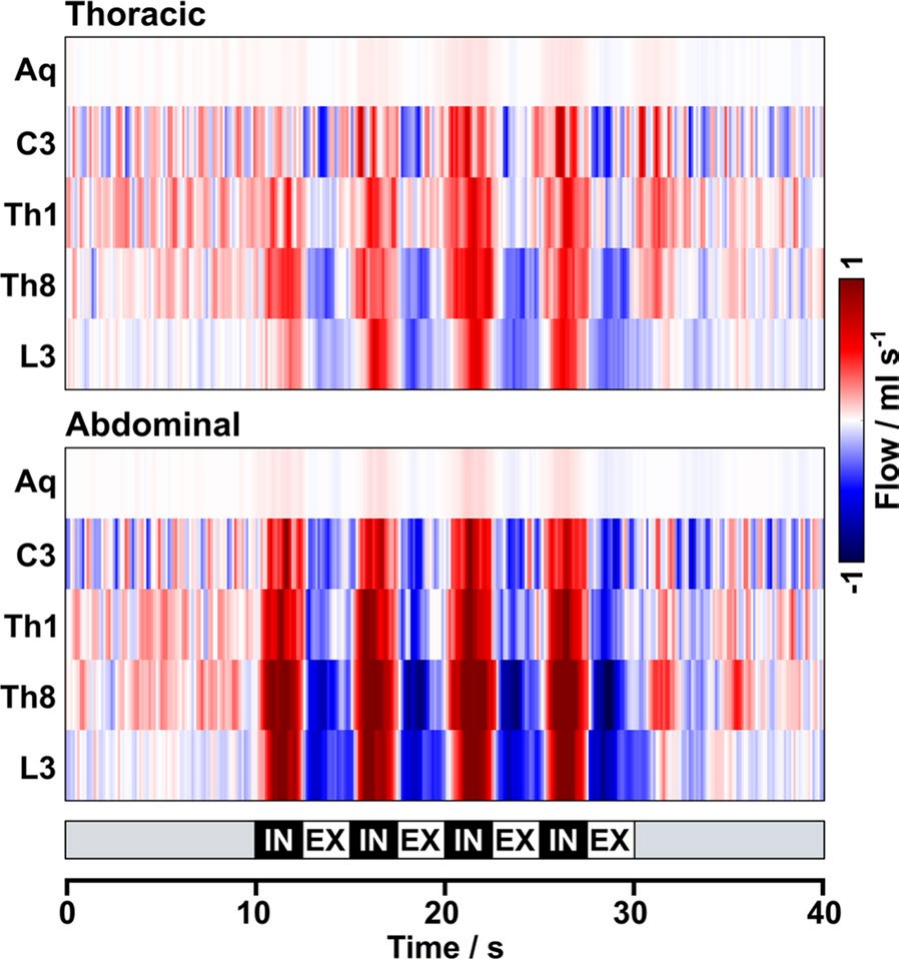
Mean CSF flow rates (ml s^−1^) during forced respiration. Mean color-coded flow rates averaged across subjects in the aqueduct as well as at spinal levels C3, Th1, Th8 and L3 for Top: forced thoracic and Bottom: forced abdominal breathing. The color scale was chosen to visualize the low flow in the aqueduct. Onset of forced inspiration prompts an increase of CSF flow in upward direction (red) at all locations, while downward movement (blue) prevails during expiration at all spinal levels. Forced abdominal breathing consistently causes higher flow rates compared to thoracic breathing

The resulting CSF dynamics in response to the two breathing types are summarized in Fig. 3. The upper and lower part represent color-coded flow rates (ml s^−1^) averaged across all 18 subjects during thoracic and abdominal breathing, respectively. The results uniformly indicate cranially directed CSF flow (red) with every forced inspiration at all levels. During exhalation CSF follows a caudal movement (blue) to a variable extent but most pronounced in the lower thoracic region (Th8). Abdominal breathing clearly elicits higher flow rates during forced respiration compared to thoracic breathing. Periods of uncontrolled “normal breathing” before and after forced respiration revealed no clear trend. Flow rates in the aqueduct remained constantly low and showed no clear differences between breathing types. During every forced inspiration small positive flow values (faint red) could be measured. Concomitant forced expiration yielded even smaller though mostly negative (faint blue) values.

**Fig. 4:**
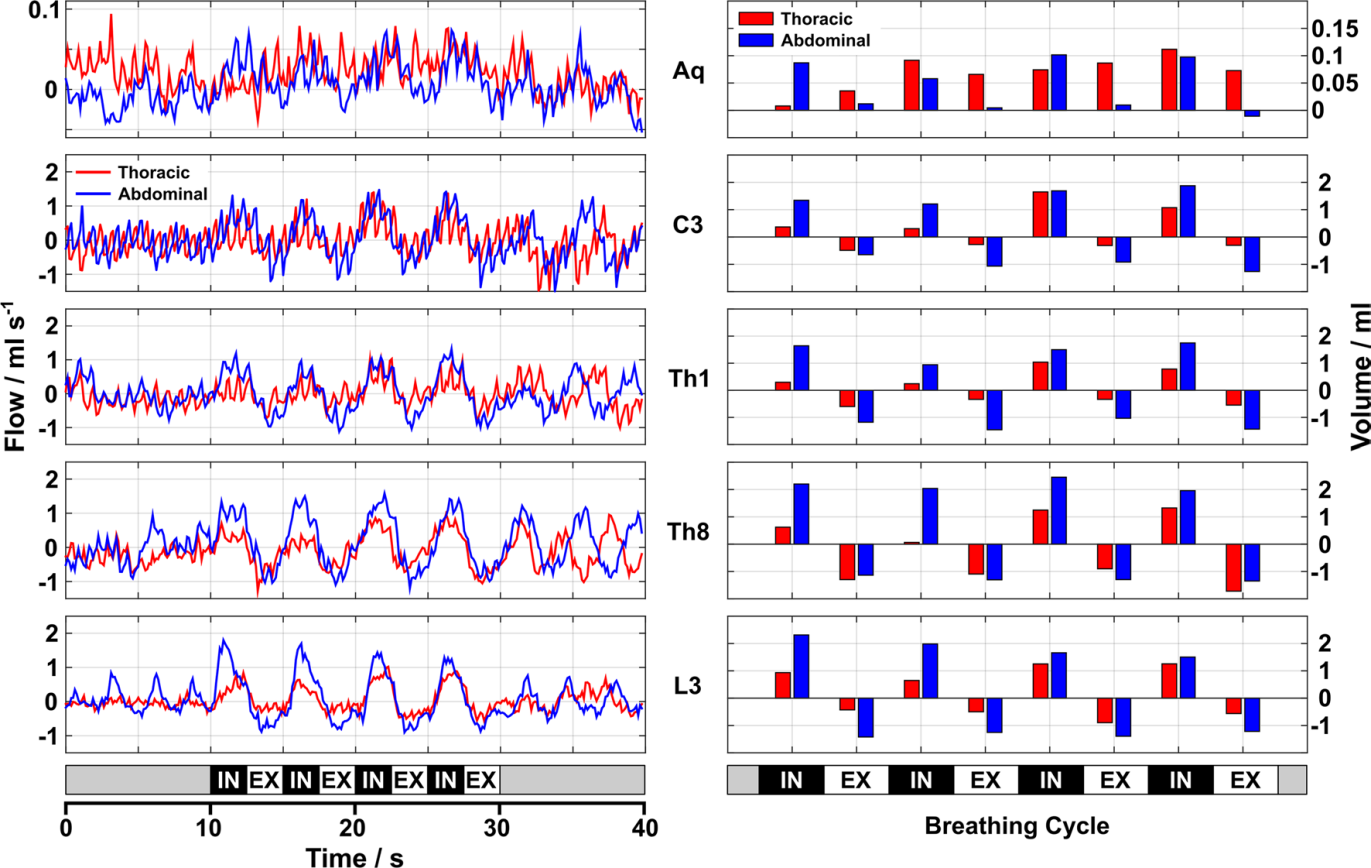
CSF dynamics during thoracic and abdominal breathing. Left: CSF flow in aqueduct and spinal canal (subject #16) during (red) thoracic or (blue) abdominal breathing. Right: CSF volumes during four cycles of forced respiration. The onset of forced inspiration leads to a distinct increase of upward CSF flow which is reversed during expiration. In spinal canal, abdominal breathing causes higher flow rates and volumes, which is not seen in the aqueduct (small flow rates and volumes). *Aq* aqueduct, *C3* cervical level 3, *Th1/Th8* thoracic levels 1 and 8, *L3* lumbar level 3, *IN* inspiration, *EX* expiration

Figure 4 illustrates the CSF dynamics during the breathing protocol for one representative subject (#16). The left part displays flow rates (ml s^−1^) within the aqueduct and spinal canal for thoracic (red) and abdominal breathing (blue). In both cases forced inspiration elicited prompt and distinct upward CSF flow in all locations, while exhalation led to a reversal of CSF flow at all spinal levels, in particular at L3, Th8, and Th1. Again, in the aqueduct CSF flow rates were very low due to the narrowness of the canal and no clear downward directionality could be identified in this particular subject. Cardiac-related flow represents a small continuous component at L3 and Th8 with increasing relative influence at C3 and aqueduct.

The right part of Fig. 3 depicts the corresponding CSF flow volumes for every forced inspiration (2.5 s) and expiration (2.5 s). Inspiratory flow volumes refer to upward motion with highest (positive) values at Th8 during abdominal breathing. Expiratory flow volumes were directed downwards in all spinal locations and breathing conditions, but clearly more pronounced for abdominal compared to thoracic breathing. In the aqueduct much smaller expiratory flow volumes were elicited, while positive values again indicate upward movement towards the 3rd ventricle.

**Fig. 5:**
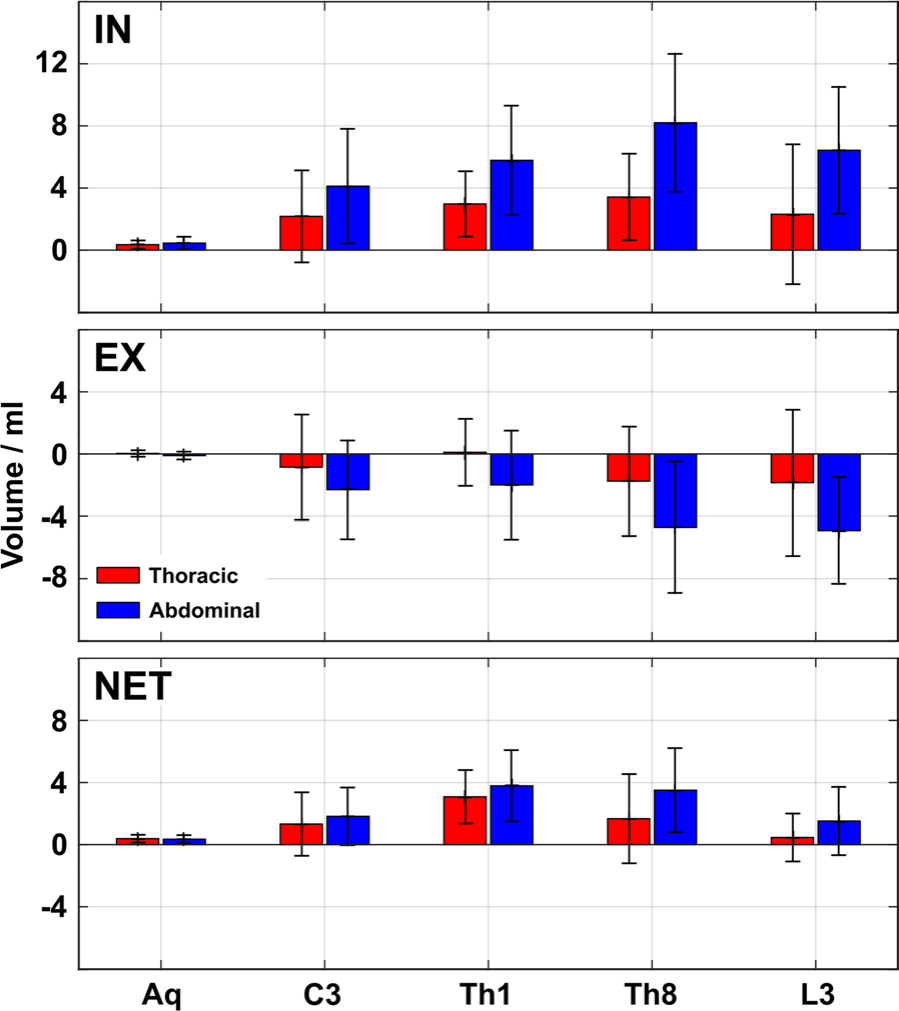
CSF net volumes during forced respiration. Mean CSF flow volumes averaged across 18 subjects and 4 cycles of forced thoracic (red) and abdominal (blue) Top: inspiration and Middle: expiration. Positive inspiratory CSF flow volumes indicate upward fluid movement which was more pronounced during abdominal breathing. Negative downward flow volumes during forced expiration varied, but showed a more uniform behavior during abdominal respiration. The large standard deviations during forced thoracic expiration indicate pronounced inter-individual differences. Bottom: CSF net volumes point upwards at all locations, again more distinct during abdominal breathing. *IN* inspiration, *EX* expiration, *NET* CSF net volumes

Mean CSF flow volumes averaged across subjects and four cycles of forced inspiration and expiration, respectively, are represented in Fig. 5. Additional file 5: Table S1 provides corresponding quantitative values for each subject. Forced inspiration of both breathing types (Fig. 5, top) generated positive values representative for an upward CSF movement in all locations. In the spinal canal higher flow volumes were reached during forced abdominal inspiration. During exhalation (Fig. 5, middle) flow volumes were reversed (negative values) at all levels and for both breathing types, although flow volumes were again larger during forced abdominal breathing. In the aqueduct forced expiration elicited nearly no flow. The CSF net flow volumes averaged over 20 s of forced respiration (Fig. 5, bottom) yielded positive values in all locations and regardless of breathing type. While most prominent effects were seen at spinal levels C3, Th1 and Th8, the aqueduct again yielded very small net movement upward into the brain.

Evaluation of the individual ROIs as a function of time for all 18 subjects revealed variable sizes between measurement positions, but no distinct or consistent changes over time during both types of forced respiration (see Additional file 6: Figure S3). These results confirm that only changes of CSF flow velocities (see minimal and maximal ranges in Additional file 7: Table S2) evoke alterations of CSF volumes and not variations in ROI sizes which are in line with previous studies [10].

## Discussion

In agreement with previous findings [10] forced inspiration elicited a distinct upward surge of CSF in the entire fluid system from the lumbar region up to the aqueduct regardless of the breathing type. In contrast, and except for the aqueduct, forced expiration led to reversed, downward flow albeit to varying extent and more pronounced in the lower regions of the spinal canal.

Differences between breathing conditions emerged exclusively for spinal CSF movement, where abdominal breathing was associated with larger flow volumes than thoracic breathing at all levels in all subjects. Physiologically, deep thoracic and abdominal breathing exert diverging muscle groups. During thoracic breathing, muscle groups of the bony rib cage such as the intercostal muscles mainly extend the anteroposterior diameter of the thorax and thus accomplish breathing by elevation of the ribs, while the diaphragm follows more passively. In contrast, abdominal breathing actively utilizes the diaphragm as the most powerful inspiratory muscle [12]. Its intense contraction during forced inhalation lowers the diaphragmatic dome and results in maximal widening of the lower thoracic cavity (e.g., compare Fig. 2, Additional file 3: Video S1 and Additional file 4: Video S2). Accordingly, changes of the intrathoracic volume and in turn the intrathoracic pressure are greater during abdominal breathing compared to thoracic breathing [14]. The rapid adjustments of CSF flow during forced breathing might be explained by prompt transmission of intrathoracic and intraabdominal pressure changes via abundant connections of the paravertebral venous plexus through the intervertebral foramina to the epidural spaces and their venous plexus therein [24, 25]. The resulting CSF net flow volumes are similar for both breathing types, but also characterized by marked inter-subject variability which impedes further statistical analysis. This observation is consistent with our previous results and other studies of healthy subjects and patients [26, 27].

The finding of pulsatile fluid movements parallel to respiration is in line with our previous studies, except for a lack of downward flow at Th8 [10]. This may be explained by past instructions to direct respiratory excursions towards a respiration belt at the level of the sternum which results in less defined contributions from abdominal and thoracic breathing. Sagittal views onto thorax and diaphragm (compare Fig. 2) clearly illustrate that the region of the lower thoracic spine yields the most striking physiological differences between breathing types. In particular, the more pronounced contraction of the diaphragm during abdominal breathing leads to a greater opening of the costodiaphragmatic recess (arrows in Fig. 2) in close proximity to spinal level Th8. Therefore, breathing performances are expected to instantaneously affect CSF dynamics in the nearby spinal canal.

The CSF flow in the aqueduct was comparatively small and revealed no distinct differences between breathing types. In full agreement with previous reports CSF movement during deep inspiration was upward, while forced expiration elicited very low or no CSF flow [9, 28]. It is tempting to speculate that the aqueduct holds a regulatory function in order to prevent irregular volume variations into the ventricles of the brain. Recent findings of differential CSF flow in the aqueduct of healthy subjects and patients with idiopathic normal pressure hydrocephalus and intracranial aneurysms may be in support of this hypothesis [27, 29].

Respiration as the dominant modulator of CSF movement has been observed in few other flow MRI studies without gating [4] or using respiratory gating at 7 T [28].

Measurements with focus on the craniocervical junction and aqueduct unanimously yielded upward CSF flow prompted by inspiration and in the reverse direction during expiration [4, 28, 30].

Takizawa et al. [31] described that the cardiac-induced CSF flow moved small distances at high velocities, while respiratory components moved slow but long distances in aqueduct and craniocervical junction thus indicating the responsiveness of the CSF system to variations in pressure. Moreover, comparing normal breathing with deep abdominal breathing, Yildiz et al. [30] found a higher contribution of the respiratory component on CSF velocity at the craniocervical junction during the latter one. Interestingly, early Doppler ultrasonography studies in infants revealed CSF dynamics directly related to respiration. The observed upward direction during inspiration and reverse direction during exhalation is in line with our present findings [32, 33]. Furthermore, Winkler explored changes of CSF flow during increased abdominal pressure applied by swift flat hand pressing onto the infants’ belly. The observed immediate effects on CSF flow again point towards its high sensitivity to rapid variations of intraabdominal pressure [34]. Remarkably, the author described an association between the cessation of flow synchronous to respiration and increasing dominance of cardiac component with evolving intracranial diseases such as progressive hydrocephalus, edema, or progressive cystic tumor [34].

## Conclusions

Spinal CSF dynamics are highly sensitive to respiratory performance and instantaneously reflect intraabdominal and intrathoracic volume and associated pressure changes. Forced inspiration and expiration therefore lead to upward and downward CSF flow in the spinal canal, respectively. Respective flow rates and volumes are much more pronounced for abdominal than for thoracic breathing, while net flow volumes for a 20 s pattern of forced respiration resulted in rather similar positive values, i.e. upward movement, for both breathing types and at all locations. On the other hand, the aqueduct not only yields much smaller flow rates and volumes, but also retains an upward movement during respiration which, in agreement with previous observations, is more pronounced during inspiration.

The ability of the CSF system to accommodate a broad physiological range of pressure conditions is of high clinical importance for patients with disturbed CSF circulation like hydrocephalus, pseudotumor cerebri and others. Real-time MRI access to quantitative CSF flow in these patients will therefore contribute to unravel underlying pathophysiological mechanisms and to open new approaches to therapeutic interventions.

## Additional files

**Additional file 1: Figure S1.** Regions-of-interest along spinal CSF space and aqueduct. Sagittal T2-weighted image of the whole spine indicating selected cross-sections for ROI placements. Aq = aqueduct; C3 = cervical level 3; Th1/Th8 = thoracic levels 1/8; L3 = lumbar level 3.

**Additional file 2: Figure S2.** Adherence to the breathing protocol. Upper part: CSF flow (ml s^−1^) (black line) closely follows movements of the abdominal wall (a.u.) (blue dotted line) (subject #18). Note the distinct amplitude increase reflecting extensive movements of abdominal wall during forced respiration. The timing of the respiratory cycles was incorrect. CSF motion correlates with breathing but not with time intervals of the protocol (at the bottom). Correlation function (orange line) was applied to correct for the shift in time. Lower part: after correcting for the error in timing. Signal of abdominal wall and corresponding CSF flow matches the timing of the protocol. IN = inspiration; EX = expiration; S.I. = signal intensity; a.u. = arbitrary units.

**Additional file 3: Video S1.** Real-time MRI of forced thoracic respiration at 30 fps. Sagittal plane through right diaphragmatic dome. The thoracic cavity enlarges during inspiration because of elevation of frontal thorax wall and lowering of the diaphragm. The space between the diaphragm and the rear thoracic wall (costodiaphragmatic recess) increases.

**Additional file 4: Video S2.** Real-time MRI of forced abdominal respiration at 30 fps. Sagittal plane through right diaphragmatic dome. Downward movement of the diaphragm is more pronounced during forced inspiration.

**Additional file 5: Table S1.** Mean CSF volumes during 4 cycles (20 s) of forced in- and expiration (ml). Mean CSF flow volumes averaged across 4 cycles of forced inspiration and expiration, respectively, for all 18 subjects. Aq = aqueduct; C3 = cervical level 3; Th1/Th8 = thoracic levels 1/8; L3 = lumbar level 3; Abd = abdomen; In = inspiration; Ex = expiration.

**Additional file 6: Figure S3.** Time courses of ROI areas (mm^2^) for CSF analysis. Color-coded mean areas averaged across subjects show no significant change over time during forced thoracic (upper part) and abdominal (lower part) breathing. Aq = aqueduct; C3 = cervical level 3; Th1/Th8 = thoracic levels 1/8; L3 = lumbar level 3; In = inspiration; Ex = expiration.

**Additional file 7: Table S2.** Minimal and maximal CSF flow velocities (cm s^−1^) during 20 s of forced in- and expiration. Minimum and maximum velocities of all 18 subjects at all levels obtained during 20 s of forced thoracic and abdominal breathing, respectively. Pos = position; Aq = aqueduct; C3 = cervical level 3; Th1/Th8 = thoracic levels 1/8; L3 = lumbar level 3.

## Abbreviations

CSF: cerebrospinal fluid
C3: cervical spine at level 3
ECG: electrocardiogram
L3: lumbar spine at level 3
MRI: magnetic resonance imaging
SD: standard deviation
Th1: thoracic spine at level 1
Th8: thoracic spine at level 8
