# Supplementary figures and images for: Spinal CSF flow in response to forced thoracic and abdominal respiration
Source: Fluids Barriers CNS. 2019 Apr 4;16:10. doi: 10.1186/s12987-019-0130-0 (PMC6449937; doi:10.1186/s12987-019-0130-0)

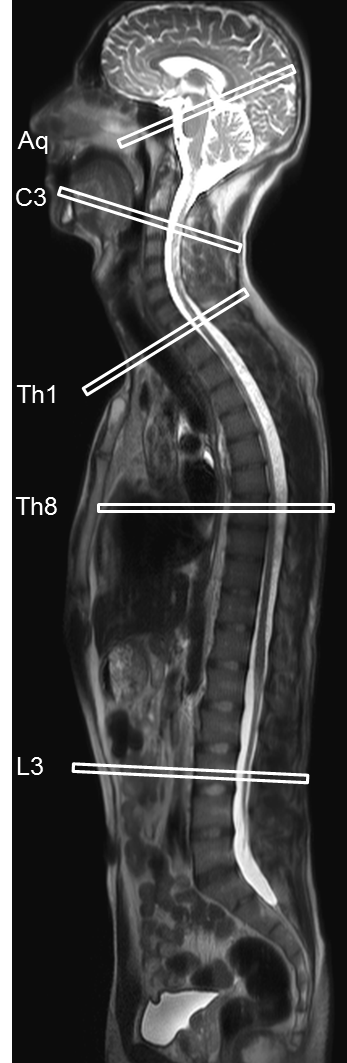

Supplement: Supplementary file 1 — Additional file 1: Figure S1. Regions-of-interest along spinal CSF space and aqueduct. Sagittal T2-weighted image of the whole spine indicating selected cross-sections for ROI placements. Aq = aqueduct; C3 = cervical level 3; Th1/Th8 = thoracic levels 1/8; L3 = lumbar level 3. [file 12987_2019_130_MOESM1_ESM.png]

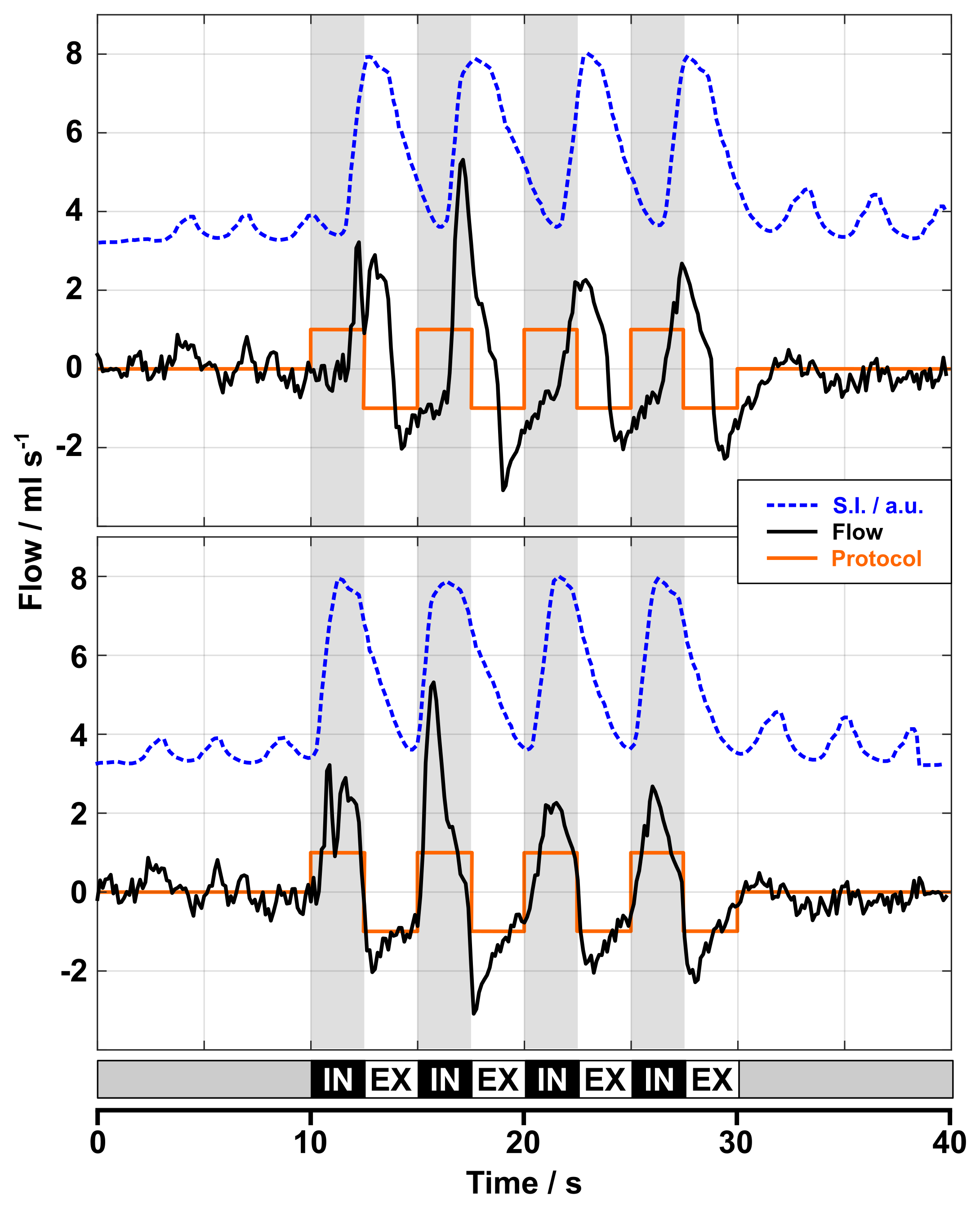

Supplement: Supplementary file 2 — Additional file 2: Figure S2. Adherence to the breathing protocol. Upper part: CSF flow (ml s−1) (black line) closely follows movements of the abdominal wall (a.u.) (blue dotted line) (subject #18). Note the distinct amplitude increase reflecting extensive movements of abdominal wall during forced respiration. The timing of the respiratory cycles was incorrect. CSF motion correlates with breathing but not with time intervals of the protocol (at the bottom). Correlation function (orange line) was applied to correct for the shift in time. Lower part: after correcting for the error in timing. Signal of abdominal wall and corresponding CSF flow matches the timing of the protocol. IN = inspiration; EX = expiration; S.I. = signal intensity; a.u. = arbitrary units. [file 12987_2019_130_MOESM2_ESM.png]

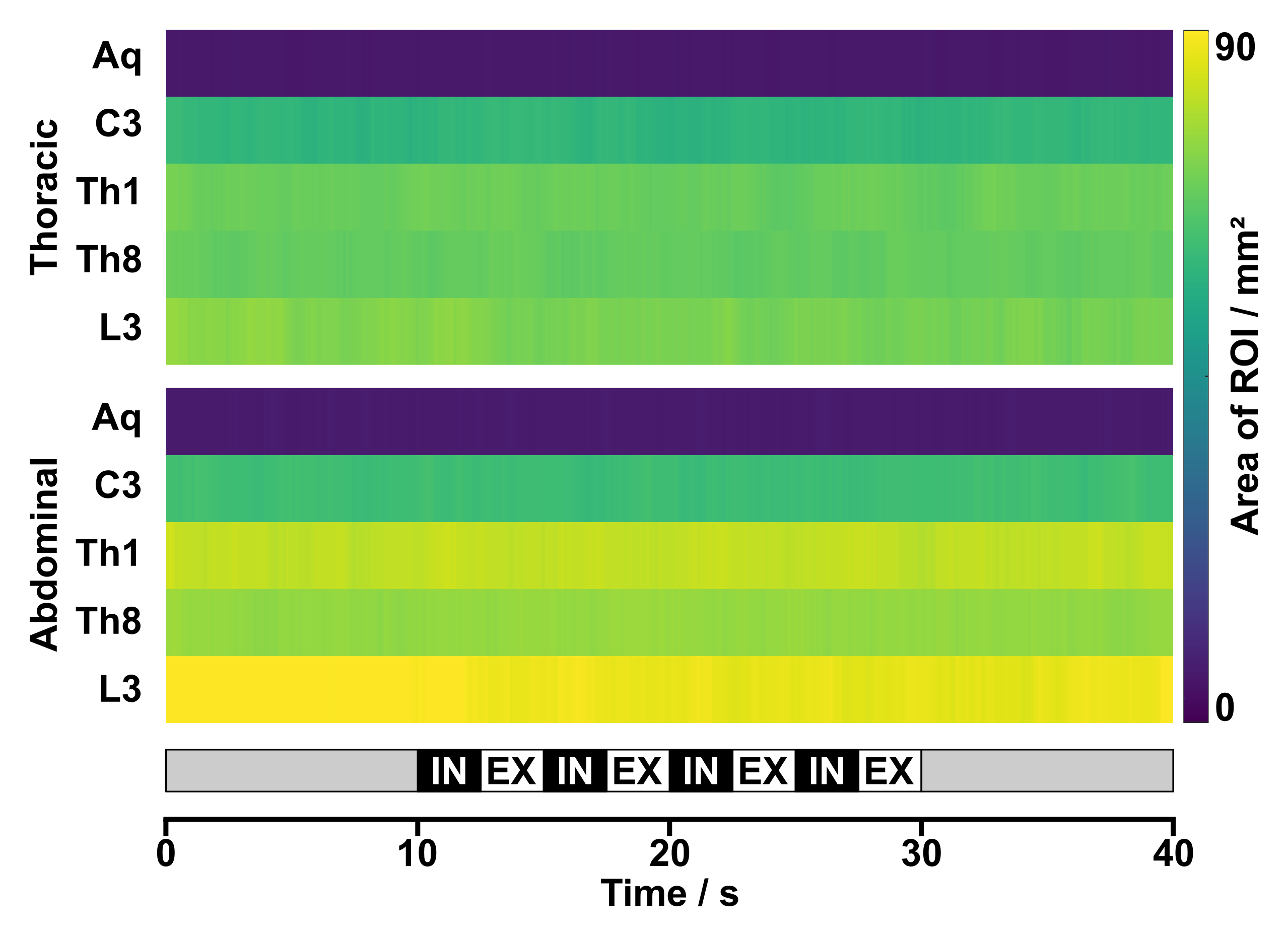

Supplement: Supplementary file 6 — Additional file 6: Figure S3. Time courses of ROI areas (mm2) for CSF analysis. Color-coded mean areas averaged across subjects show no significant change over time during forced thoracic (upper part) and abdominal (lower part) breathing. Aq = aqueduct; C3 = cervical level 3; Th1/Th8 = thoracic levels 1/8; L3 = lumbar level 3; In = inspiration; Ex = expiration. [file 12987_2019_130_MOESM6_ESM.png]
