# Supplementary material for: Spinal CSF flow in response to forced thoracic and abdominal respiration
Source: Fluids Barriers CNS. 2019 Apr 4;16:10. doi: 10.1186/s12987-019-0130-0 (PMC6449937; doi:10.1186/s12987-019-0130-0)
Supplement: Supplementary file 5 — Additional file 5: Table S1. Mean CSF volumes during 4 cycles (20 s) of forced in- and expiration (ml). Mean CSF flow volumes averaged across 4 cycles of forced inspiration and expiration, respectively, for all 18 subjects. Aq = aqueduct; C3 = cervical level 3; Th1/Th8 = thoracic levels 1/8; L3 = lumbar level 3; Abd = abdomen; In = inspiration; Ex = expiration. [file 12987_2019_130_MOESM5_ESM.docx]

**Table S1: Mean CSF volumes during 4 cycles (20 s) of forced in- and expiration (ml)**

|  | **Subject** |  | **#1** | **#2** | **#3** | **#4** | **#5** | **#6** | **#7** | **#8** | **#9** | **#10** | **#11** | **#12** | **#13** | **#14** | **#15** | **#16** | **#17** | **#18** |
| --- | --- | --- | --- | --- | --- | --- | --- | --- | --- | --- | --- | --- | --- | --- | --- | --- | --- | --- | --- | --- |
| **Aq** | **Thoracic** | **In** | 0.07 | 0.04 | 0.04 | 0.07 | 0.13 | 0.07 | 0.27 | 0.04 | n.a. | 0.09 | 0.10 | 0.11 | 0.08 | 0.02 | -0.01 | 0.07 | 0.09 | 0.23 |
|  |  | **Ex** | -0.01 | -0.01 | -0.01 | 0.02 | 0.09 | 0.00 | -0.08 | 0.02 | n.a. | -0.01 | 0.01 | 0.11 | 0.04 | 0.00 | 0.02 | 0.07 | 0.00 | -0.11 |
|  | **Abd** | **In** | 0.05 | 0.06 | 0.02 | 0.03 | 0.16 | 0.05 | 0.33 | 0.05 | n.a. | 0.07 | 0.24 | 0.14 | 0.19 | 0.04 | -0.02 | 0.09 | 0.10 | 0.33 |
|  |  | **Ex** | 0.06 | -0.04 | 0.05 | 0.03 | 0.05 | -0.02 | -0.18 | 0.00 | n.a. | 0.01 | -0.05 | -0.04 | -0.07 | -0.01 | -0.04 | 0.00 | -0.02 | -0.15 |
| **C3** | **Thoracic** | **In** | 1.23 | -0.30 | 0.74 | -0.29 | -0.29 | 0.15 | 1.80 | -0.15 | 0.73 | -0.10 | 0.43 | 0.64 | 0.26 | 0.81 | -0.05 | 0.85 | 0.79 | 2.53 |
|  |  | **Ex** | -0.99 | 0.26 | -1.43 | 0.91 | 0.65 | -0.03 | -0.92 | 0.23 | 0.06 | 0.40 | -0.40 | 0.07 | -0.03 | 0.84 | -0.28 | -0.35 | -0.13 | -2.69 |
|  | **Abd** | **In** | 0.96 | -0.53 | -0.05 | -0.10 | -0.10 | 0.93 | 2.85 | 1.22 | 0.90 | 1.11 | 1.73 | 1.49 | 1.29 | 1.25 | 0.29 | 1.53 | 0.72 | 3.05 |
|  |  | **Ex** | 0.04 | 0.26 | 0.15 | 0.23 | 0.28 | -0.81 | -1.96 | -0.64 | 0.12 | -0.20 | -1.01 | -0.95 | -1.39 | 0.17 | -0.54 | -0.97 | -0.56 | -2.57 |
| **Th1** | **Thoracic** | **In** | 0.48 | 0.11 | 0.87 | 1.21 | 0.77 | 0.02 | 1.29 | 0.57 | 0.41 | 0.40 | 1.12 | 0.65 | 0.63 | 0.58 | 0.20 | 0.59 | 1.14 | 2.33 |
|  |  | **Ex** | 0.79 | 0.39 | -0.13 | -0.18 | 0.89 | 0.30 | -0.59 | 0.37 | 0.02 | 0.50 | -0.81 | 0.24 | 0.64 | -0.23 | -0.10 | -0.45 | 0.01 | -1.18 |
|  | **Abd** | **In** | 2.29 | 1.33 | 0.76 | 0.16 | 1.43 | 0.72 | 2.04 | 1.42 | 0.94 | 1.55 | 2.20 | 1.40 | 1.72 | 0.62 | 0.74 | 1.46 | 0.98 | 4.26 |
|  |  | **Ex** | -0.34 | -0.58 | 0.09 | 0.55 | 0.91 | -0.22 | -1.06 | 0.18 | -0.05 | -0.49 | -1.32 | -0.98 | -0.12 | -0.51 | -0.22 | -1.28 | -0.30 | -3.23 |
| **Th8** | **Thoracic** | **In** | 0.45 | -0.13 | 0.51 | -0.29 | 0.44 | 0.70 | 0.68 | 1.63 | 0.61 | 1.56 | 0.85 | 0.38 | 1.09 | 0.43 | 0.90 | 0.82 | 0.89 | 2.47 |
|  |  | **Ex** | -0.10 | 0.32 | -0.37 | 0.20 | 0.17 | 0.03 | -0.07 | 0.33 | -0.82 | -0.48 | -0.97 | -0.06 | -0.12 | -0.25 | -0.21 | -1.25 | -0.50 | -3.55 |
|  | **Abd** | **In** | 2.23 | 0.88 | 1.64 | 1.15 | 0.72 | 1.27 | 5.66 | 2.78 | 2.54 | 2.38 | 2.48 | 2.87 | 1.78 | 0.66 | 1.20 | 2.16 | 1.23 | 3.07 |
|  |  | **Ex** | -0.83 | -0.70 | -1.09 | -0.53 | -0.22 | 0.21 | -3.90 | -0.80 | -0.24 | -2.29 | -2.96 | -1.73 | -0.92 | -0.31 | -0.51 | -1.27 | -0.75 | -3.22 |
| **L3** | **Thoracic** | **In** | 0.62 | 0.19 | 1.18 | -0.13 | 0.11 | -0.59 | -0.11 | 0.23 | 0.13 | -0.11 | 0.36 | 0.19 | 1.14 | 0.37 | 0.02 | 1.02 | 0.97 | 4.79 |
|  |  | **Ex** | 0.10 | -0.27 | -0.83 | 0.04 | -0.14 | -0.16 | 0.01 | -0.01 | 0.07 | 0.03 | -0.52 | 0.20 | -0.11 | -0.17 | 0.04 | -0.60 | -0.85 | -5.14 |
|  | **Abd** | **In** | 3.08 | 0.91 | 1.71 | 0.71 | 0.79 | -0.04 | 2.64 | 1.07 | 0.52 | 1.29 | 3.53 | 2.01 | 1.75 | 1.42 | 0.63 | 1.87 | 1.38 | 3.67 |
|  |  | **Ex** | -1.33 | -0.78 | -0.94 | -0.61 | -0.58 | -0.46 | -2.08 | -0.26 | -0.06 | -1.40 | -2.68 | -2.36 | -0.63 | -1.02 | -1.07 | -1.32 | -1.11 | -3.38 |
