# Supplementary material for: Spinal CSF flow in response to forced thoracic and abdominal respiration
Source: Fluids Barriers CNS. 2019 Apr 4;16:10. doi: 10.1186/s12987-019-0130-0 (PMC6449937; doi:10.1186/s12987-019-0130-0)
Supplement: Supplementary file 7 — Additional file 7: Table S2. Minimal and maximal CSF flow velocities (cm s−1) during 20 s of forced in- and expiration. Minimum and maximum velocities of all 18 subjects at all levels obtained during 20 s of forced thoracic and abdominal breathing, respectively. Pos = position; Aq = aqueduct; C3 = cervical level 3; Th1/Th8 = thoracic levels 1/8; L3 = lumbar level 3. [file 12987_2019_130_MOESM7_ESM.docx]

**Table S2: Ranges of minimal and maximal CSF flow velocities during 20 s of forced in- and expiration**

| **Pos** | **Range of minimum velocities (cm s^-1^)** | **Range of maximum velocities (cm s^-1^)** |
| --- | --- | --- |
| **Thoracic breathing** | | |
| **Aq** | -3.8 − -1.2 | 2.5 − 4.3 |
| **C3** | -8.8 − -6.6 | 5.9 − 9.5 |
| **Th1** | -5.4 − -2.9 | 3.8 − 5.6 |
| **Th8** | -11.9 − -8.7 | 6.5 − 8.2 |
| **L3** | -9.6 − -3.9 | 3.2 − 13.9 |
| **Abdominal breathing** | | |
| **Aq** | -3.4 − -1.6 | 2.5 − 4.4 |
| **C3** | -7.9 − -5.6 | 6.7 − 7.8 |
| **Th1** | -5.4 − -4.1 | 4.7 − 9.9 |
| **Th8** | -10.1 − -6.5 | 6.8 − 11.4 |
| **L3** | -5.8 − -3.8 | 5.5 − 8.5 |
